# Uremic Encephalopathy Presented as Stroke: The Value of Lentiform Fork Sign

**DOI:** 10.7759/cureus.74339

**Published:** 2024-11-24

**Authors:** Fahad Albadr, Abdulmohsen S Alqadeeb, Walid s Alzaid, Faisal A Alalwan, Yazeed A Alomar, Muhannad A Alomar, Abdullah M Bafarat

**Affiliations:** 1 Radiology and Medical Imaging/Neuroradiology, King Saud University Medical City, Riyadh, SAU; 2 College of Medicine, King Saud University, Riyadh, SAU; 3 Department of Radiology and Medical Imaging, King Abdulaziz Medical City, Riyadh, SAU

**Keywords:** basal ganglia hyperintensities, case report, lentiform fork sign, stoke, uremic encephalopathy

## Abstract

Uremic encephalopathy (UE) is a neurological complication of renal failure characterized by cognitive dysfunction and movement abnormalities. A novel radiologic sign termed the "lentiform fork sign" has been identified in patients with UE and metabolic acidosis. This sign manifests as bilateral symmetrical hyperintensities in the basal ganglia, bordered by a hyperintense rim on magnetic resonance imaging (MRI), particularly on T2-weighted and fluid-attenuated inversion recovery (FLAIR) sequences. The basal ganglia, highly metabolically active structures, are prone to damage from metabolic derangements, toxins, and systemic conditions.

We present a 56-year-old male with a history of chronic kidney disease and diabetes mellitus, maintained on hemodialysis and metformin. The patient presented with acute neurological symptoms, including slurred speech, left-sided weakness, and dysarthria. Brain MRI revealed bilateral basal ganglia hyperintensities on T2-weighted images, consistent with the lentiform fork sign. Laboratory investigations showed elevated serum urea and metabolic acidosis, suggestive of uremic encephalopathy. The patient’s condition improved following dialysis, leading to partial resolution of neurological symptoms.

## Introduction

Uremic encephalopathy (UE) is a complex neurological condition that arises due to the accumulation of uremic toxins in individuals with chronic kidney disease (CKD). Basal ganglia (BG) are characteristically involved in UE [1]. The pathophysiological mechanisms underlying uremic encephalopathy are multifaceted. Research indicates that uremic toxins play a pivotal role in the development of cognitive dysfunction and other neurological complications associated with CKD. These toxins can induce oxidative stress, endothelial dysfunction, and renal interstitial fibrosis, which contribute to the deterioration of cognitive function [2]. Notably, the role of colonic bacteria as a source of these toxins has been emphasized, suggesting that microbial dysbiosis may exacerbate uremic symptoms [3].

Moreover, patients with CKD exhibit a significantly higher risk of cognitive impairment compared to the general population. Lower glomerular filtration rates (GFR) and the presence of albuminuria are closely linked to cognitive decline, indicating that renal function directly influences neurological health [4]. This relationship underscores the importance of managing kidney health to mitigate cognitive deterioration. Uremic encephalopathy manifests through various neurological symptoms, including cognitive impairment, delirium, and acute encephalopathy. A significant proportion of patients with severe CKD experience acute encephalopathy, highlighting the acute nature of this complication [5].

The results of magnetic resonance imaging (MRI) reveal a variety of distinct characteristics, mostly pertaining to three primary brain regions: first, BG; second, cortical and subcortical regions; and third, multiple white matter regions [6]. Lately, visualization of the lentiform fork sign (LFS) on MRI has been becoming acknowledged as a valuable imaging indicator for an accurate diagnosis of UE. The hyperintense rim delineating the putamen on the T2-weighted imaging (T2WI) sequence of MRI in LFS reflects the edema of these regions [6]. While the lentiform fork sign is commonly associated with UE, it is not exclusive to this condition. Differential diagnoses that also exhibit similar MRI findings include Wilson's disease and Systemic Lupus Erythematosus (SLE). In the cases reported by the studies, patients with Wilson's disease and SLE did not exhibit metabolic acidosis, a key clinical feature often observed in uremic encephalopathy. Accurate recognition and distinction of these conditions are essential as they require different management strategies compared to UE [7,8]. The lentiform fork sign, observable in various clinical scenarios, becomes more pronounced under certain conditions that exacerbate metabolic stress in the basal ganglia. These include diabetes mellitus type 2, methanol poisoning, and ethylene glycol poisoning. In these cases, underlying metabolic disturbances and the accumulation of toxic metabolites increase the susceptibility of the basal ganglia to damage, thereby enhancing the prominence of the lentiform fork sign on imaging [9]. Optimizing dialysis frequency, correcting metabolic acidosis in patients with metabolic acidosis, managing sepsis, stabilizing blood glucose levels, and discontinuing nephrotoxic medications typically result in complete or partial resolution of clinical symptoms and radiological abnormalities [10].

## Case presentation

A 56-year-old male with a history of end-stage renal disease (ESRD) on maintenance hemodialysis, type 2 diabetes mellitus on metformin presented to the Emergency Department with fatigue associated with nausea, mild confusion, and decreased concentration, increased left-sided weakness with numbness from his baseline and increased dysarthria from his baseline after coming back from a dialysis session. His past medical history was significant for an ischemic stroke five years prior, which manifested as left hemiparesis and dysarthria, with residual weakness in the lower limb. On examination, his vital signs were: temperature 36.8°C, blood pressure 162/96 mmHg, heart rate 106 beats per minute (bpm), and oxygen saturation 95% on room air. Pupils were bilaterally reactive to light. The patient exhibited left-sided facial deviation, left upper limb weakness, and decreased sensation. There were no signs of neck rigidity, and both Kernig's and Brudzinski's signs were negative. The rest of the systemic examination was unremarkable.

Laboratory investigations were within normal range except for deranged renal function tests, with a creatinine level of 846 µmol/L. Serum electrolytes revealed hyponatremia (sodium 130 mEq/L). Liver function tests, thyroid function tests, and coagulation profiles were within normal limits (Table 1).

**Table 1:** Baseline laboratory tests at the time of presentation Hgb: hemoglobin, PLT: platelets, Na: sodium, Cl: chloride, K: potassium, Bilirubin: bilirubin, ALT: alanine aminotransferase, ALP: alkaline phosphatase, HCO_3_: bicarbonate, Pco_2_: partial pressure of carbon dioxide, Po_2_: partial pressure of oxygen, HbA1c: hemoglobin A1c.

Laboratory tests	Results	Normal range
Hgb	11 g/dL	13.8–17.2 g/dL
PLT	288 x 10^9^ /L	150,000–450,000 /L
Na	130 mEq/L	135–145 mEq/L
Cl	91 mEq/L	98–106 mEq/L
K	4 mEq/L	3.5–5.0 mEq/L
Bilirubin	7.4 µmol/L	1.7–20.5 µmol/L
ALT	13 units/L	7–56 units/L
ALP	66 units/L	44–147 units/L
pH	7.35	7.35–7.45
HCO_3_	22 mEq/L	22–28 mEq/L
Pco_2_	44 mmHg	35–45 mmHg
Po_2_	90 mmHg	75–100 mmHg
HbA1c	6.9%	4.0–5.6%

**Figure 1:**
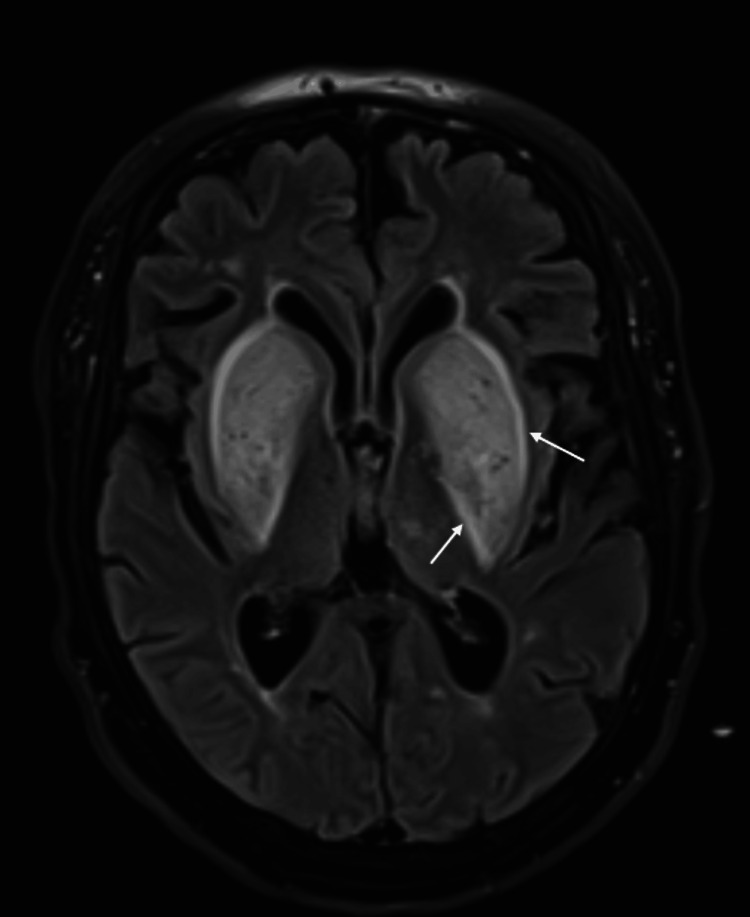
Axial T2-weighted FLAIR imaging of the brain Axial T2-weighted fluid-attenuated inversion recovery (FLAIR) imaging demonstrates bilateral symmetrical swollen lentiform nuclei with a hyperintense rim delineating the lentiform nucleus with a typical fork-like appearance representing the “lentiform fork" sign (arrow).

**Figure 2:**
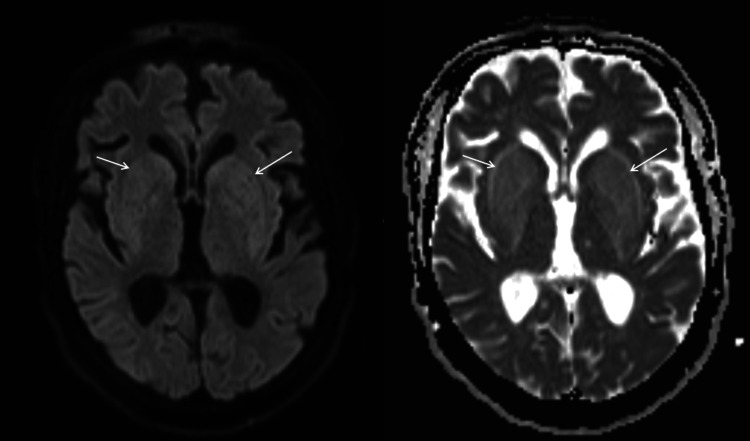
DWI and ADC map images of the brain Diffusion-weighted imaging (DWI) and apparent diffusion coefficient (ADC) map images demonstrate bilateral mild high signal intensity with no diffusion restriction.

An MRI of the brain was performed, revealing bilateral basal ganglia hyperintense T2 and fluid-attenuated inversion recovery (FLAIR) signals, with a pattern consistent with the “lentiform fork sign” seen in uremic encephalopathy (Figure 1). No diffusion restriction was observed on diffusion-weighted imaging (DWI) (Figure 2).

Permissive hypertension was allowed for 24 hours with a target blood pressure of up to 200/105 mmHg. Metformin was held, and an insulin sliding scale was started for glycemic control. The patient was admitted for further management. The patient's neurological symptoms gradually improved over three weeks, coinciding with the stabilization of his metabolic profile, including dialysis sessions conducted in the hospital. He was ultimately diagnosed with uremic encephalopathy based on his clinical presentation of extrapyramidal symptoms, laboratory results, and imaging findings.

## Discussion

We report a diabetic patient with end-stage renal disease (ESRD) on hemodialysis, metabolic acidosis, and uremic encephalopathy, who presented with neurological symptoms and the rare “lentiform fork” sign on T2-weighted brain MRI. This sign found in the basal ganglia, an area vulnerable to toxins and metabolic products, correlated with the patient’s clinical diagnosis of diabetic uremic syndrome [11].

The MRI findings in uremic encephalopathy (UE) typically present with distinct patterns. In basal ganglia (BG) lesions, there are bilateral, expansile, and symmetric lesions with increased signal intensity on T2-weighted and FLAIR images, often exhibiting the characteristic lentiform fork sign (LFS). Cortical lesions show high signal intensity on T2-weighted and FLAIR images, predominantly affecting the frontal and parieto-occipital lobes. Diffusion-weighted imaging (DWI) reveals varied levels of restricted diffusion, suggesting a combination of cytotoxic and/or vasogenic edema.

According to Rosner et al. [12], uremic encephalopathy potentially results from the build-up of uremic waste products, hormonal dysregulation, alterations in electrolyte and acid-base homeostasis, as well as changes in endothelial responsiveness, blood-brain barrier permeability, and inflammation. The diagnosis is often made retrospectively based on symptom improvement following dialysis or transplantation, as there are no definitive clinical, laboratory, or imaging findings.

Our case findings correlate with the clinical symptoms, reinforcing the diagnosis of uremic encephalopathy. In the acute setting, it is crucial to consider various differential diagnoses alongside uremic encephalopathy; these include hypoglycemia, cerebral hypoxic encephalopathy, carbon monoxide poisoning, encephalitis, osmotic myelinolysis, toxin exposure, vascular causes, and illicit drug use [13]. However, the patient’s clinical improvement after multiple dialysis sessions supports the diagnosis of uremic encephalopathy. The consideration of these differential diagnoses highlights the importance of a comprehensive clinical evaluation to accurately determine the underlying cause of the patient’s neurological symptoms.

Metformin is a very common medication for diabetes mellitus type 2, but in patients with an estimated glomerular filtration rate (eGFR) <30 mL/min/1.73 m², it can be contraindicated due to an increased risk of lactic acidosis. Kumar and Goyal [14] hypothesized that metabolic acidosis is a key factor in the pathogenesis of clinical symptoms and MRI findings associated with uremic encephalopathy. Several cases have reported that discontinuing metformin led to the reversibility of neurological conditions. For instance, Ishizaki et al. [15] noted that clinical symptoms gradually appeared after starting metformin treatment, suggesting a link between the drug and the development of encephalopathy. In contrast, Alhusseini et al. [16] observed that although uremic encephalopathy is commonly associated with metformin usage, the patient in his study did not use metformin, suggesting that uremic encephalopathy can occur independently of metformin. Additionally, Sakurai and Nishida [17] pointed out that uremic syndrome and metformin-induced encephalopathy are clinically similar and may share a common underlying pathology, such as metabolic acidosis.

## Conclusions

The identification of radiological findings, particularly the lentiform fork sign on MRI, is critical in the early and accurate diagnosis of uremic encephalopathy in patients with metabolic acidosis and chronic kidney disease. This imaging feature offers important evidence of basal ganglia involvement, significantly impacting clinical treatment by leading to prompt interventions like dialysis and the adjustment of metabolic imbalances. This case emphasizes the importance of recognizing metabolic acidosis and evaluating medications such as metformin in patients with advanced kidney disease, since the discontinuation of these medications may result in neurological improvement. Although the lentiform fork sign can aid in diagnosis, its absence does not rule out uremic encephalopathy. The long-term effects of ongoing MRI findings are still unclear, highlighting the need for further research to assess their prognostic significance.
